# The Role of AP-1 Transcription Factors in Plasma Cell Biology and Multiple Myeloma Pathophysiology

**DOI:** 10.3390/cancers13102326

**Published:** 2021-05-12

**Authors:** Fengjuan Fan, Klaus Podar

**Affiliations:** 1 Institute of Hematology, Union Hospital, Tongji Medical College, Huazhong University of Science and Technology, Jiefang Avenue 1277, Wuhan 430022, China; fengjuan_fan@hust.edu.cn; 2 Department of Internal Medicine II, University Hospital Krems, Mitterweg 10, 3500 Krems an der Donau, Austria; 3 Molecular Oncology and Hematology Unit, Karl Landsteiner University of Health Sciences, Dr.-Karl-Dorrek-Strasse 30, 3500 Krems an der Donau, Austria

**Keywords:** activator protein 1 (AP-1), transcription factor (TF), plasma cell (PC), multiple myeloma (MM), bone marrow (BM), microenvironment

## Abstract

**Simple Summary:**

As convergence points of signaling cascades, transcription factors (TFs) play a crucial role in cell physiology, including B cell differentiation, and are deregulated in solid and hematologic malignancies, including multiple myeloma (MM), a malignant clonal plasma cell proliferative disorder. In particular, there is accumulating evidence that aberrant gene expression programs induced by the Activator Protein-1 (AP-1) TF family are associated with MM cell growth, survival, migration, drug resistance, bone marrow angiogenesis and bone disease. Therefore AP-1 TFs, which have been deemed as “undruggable” until most recently, represent appealing targets for novel therapeutic approaches. Indeed, strategies to target TFs such as AP-1 emerge among today’s most promising anti-MM therapies.

**Abstract:**

Multiple myeloma (MM) is an incurable hematologic malignancy characterized by the clonal expansion of malignant plasma cells within the bone marrow. Activator Protein-1 (AP-1) transcription factors (TFs), comprised of the JUN, FOS, ATF and MAF multigene families, are implicated in a plethora of physiologic processes and tumorigenesis including plasma cell differentiation and MM pathogenesis. Depending on the genetic background, the tumor stage, and cues of the tumor microenvironment, specific dimeric AP-1 complexes are formed. For example, AP-1 complexes containing Fra-1, Fra-2 and B-ATF play central roles in the transcriptional control of B cell development and plasma cell differentiation, while dysregulation of AP-1 family members c-Maf, c-Jun, and JunB is associated with MM cell proliferation, survival, drug resistance, bone marrow angiogenesis, and bone disease. The present review article summarizes our up-to-date knowledge on the role of AP-1 family members in plasma cell differentiation and MM pathophysiology. Moreover, it discusses novel, rationally derived approaches to therapeutically target AP-1 TFs, including protein-protein and protein-DNA binding inhibitors, epigenetic modifiers and natural products.

## 1. Introduction

First described in the 1980′s [1,2,3,4,5], members of the Activator Protein-1 (AP-1) transcription factor (TF) family contain the characteristic basic leucine zipper (bZIP) domain, which enables dimer formation via a stretch of hydrophobic leucines, and facilitates DNA interaction via positively charged amino acids. AP-1 family members include the JUN (c-Jun, JunB and JunD), FOS (c-Fos, FosB, Fra-1 and Fra-2), ATF (ATF2, ATF3/LRF1, ATF4, ATF5, ATF6B, ATF7, B-ATF, B-ATF2, B-ATF3, JDP1 and JDP2) and MAF (MafA, MafB, c-Maf, Nrl and MafF/G/K) multigene subfamilies [6]. While Jun proteins heterodimerize or homodimerize with members of their own subfamily, Fos proteins must heterodimerize. Depending on their composition, AP-1 TFs bind to the TPA-response element (TRE) [5′-TGA (C/G) TCA-3′] and, with lower affinity, to the cAMP response element (CRE) [5′-TGA CG TCA-3′], which is almost identical to TRE. Specifically, Jun: Jun dimers and Jun: Fos dimers preferentially bind to TRE and CRE, but also to variant sequences within the DNA; ATF-containing Jun: ATF and ATF: ATF dimers preferentially bind CRE; and MAF-containing dimers bind either to MAF-recognition element (MARE) I [5′-TGC TGA (C/G) TCA GCA-3′] or to MARE II [5′-TGC TGA CG TCA GCA-3′], extensions of TRE and CRE sequences [1]. In addition, AP-1 dimers interact with non-bZIP proteins including CBP/p300, p65/NFκB and Rb. AP-1 activity is induced by a multitude of intrinsic and extrinsic stimuli and environmental insults including cytokines, growth factors, direct cell-cell and cell-extracellular-matrix interactions, hormones, phorbol esters, UV radiation as well as viral and bacterial infections. It is predominantly regulated via MAPK-, PI3K- and NFκB- dependent transcription, but also via post-translational phosphorylation, mRNA turnover and protein stability [1,7,8]. Ultimately, these events determine specific transcriptional programs.

Accounting for ~10% of hematologic malignancies, Multiple Myeloma (MM) is characterized by the clonal expansion of malignant plasma cells (PCs) within the bone marrow (BM) and the abnormal increase of monoclonal paraprotein, leading to specific end-organ damage, including hypercalcemia, renal failure, anemia and lytic bone lesions (CRAB criteria) [9]. The development of MM is initiated from a pre-malignant, asymptomatic stage called Monoclonal Gammopathy of Undetermined Significance (MGUS), and a more advanced pre-malignant, asymptomatic stage called Smoldering MM (SMM), due to cytogenetic alterations in post-germinal center (GC) PCs. During the evolution of MGUS or SMM into MM and ultimately PC leukemia (PCL), additional genetic aberrations as well as the supportive BM microenvironment play pivotal roles [10,11]. The incidence of MGUS is >3% of the population over the age of 50, with a progression rate of 1% per year to MM; whereas SMM transforms to MM at a rate of ~10% per year during the first five years after diagnosis. As primary genetic events, approximately 40% of MM patients harbor trisomies of chromosomes, ~30% have immunoglobulin (Ig) heavy chain (IgH) translocations and ~15% have both trisomies and IgH translocations. The IgH locus is located on chromosome 14q32, and the translocations and genes affected include t(4;14)(p16;q32) (*FGFR3* and *MMSET*), t(6;14)(p21;q32) (*CCND3*), t(11;14)(q13;q32) (*CCND1*), t(14;16)(q32;q23) (*c-MAF*) and t(14;20)(q32;q11) (*MAFB*). Secondary genetic events include gains and deletions of chromosomes, global hypomethylation, mutations and secondary translocations t(8;14)(q24;q32) (*MYC*). Specifically, high-risk MM is characterized by the presence of gain of chromosome 1q, deletion of chromosome 17p (del(17p)), t(4;14), t(14;16), t(14;20) or p53 mutations [9,12]. Despite therapeutic advances including the introduction of ImmunoModulatory Drugs (IMiDs), proteasome inhibitors (PIs), monoclonal antibodies and most recently selinexor, a Selective Inhibitor of Nuclear Export (SINE) that binds and inactivates exportin-1 (XPO1) [13], the B Cell Maturation Antigen (BCMA) targeting antibody-drug-conjugate (ADC) belantamab-mafodotin [14], and BCMA-directed CAR-T cells [9], the management of MM remains challenging, mainly due to the development of drug resistance. Therefore, the identification of novel therapeutic targets and the development of derived anti-MM treatment strategies are urgently needed.

Our increasing knowledge of B cell differentiation and resultant generation of normal PCs have been fundamental to understand how these processes are deranged in MM cells [15]. PCs that undergo IgH switch recombination home to the BM, where they occupy special survival niches, and become long-lived PCs [16]. Besides their central role in many, if not all, physiologic processes, including PC differentiation, deregulation of AP-1 TFs has been implicated in solid and hematologic malignancies, including MM [17]. Deregulation of TFs contributes to MM pathogenesis through: (1) direct TF modifications (e.g., mutations); (2) intrinsic genetic alterations or extrinsic stimuli within the BM microenvironment that trigger signaling pathway-mediated TF activation or inhibition; (3) epigenetic changes in DNA methylation, histone modifications and non-coding RNAs; and (4) TF dependency on prolonged oncogene activity (“oncogenic addiction”) [11,18,19,20,21,22].

The present review article will comprehensively summarize our up-to-date knowledge on the critical role of AP-1 TFs in PC differentiation and MM pathophysiology. Moreover, we will discuss novel, rationally derived strategies to therapeutically target AP-1 TFs, including protein-protein and protein-DNA binding inhibitors, epigenetic modifiers and natural products.

## 2. AP-1 in Plasma Cell Biology

**Figure 1:**
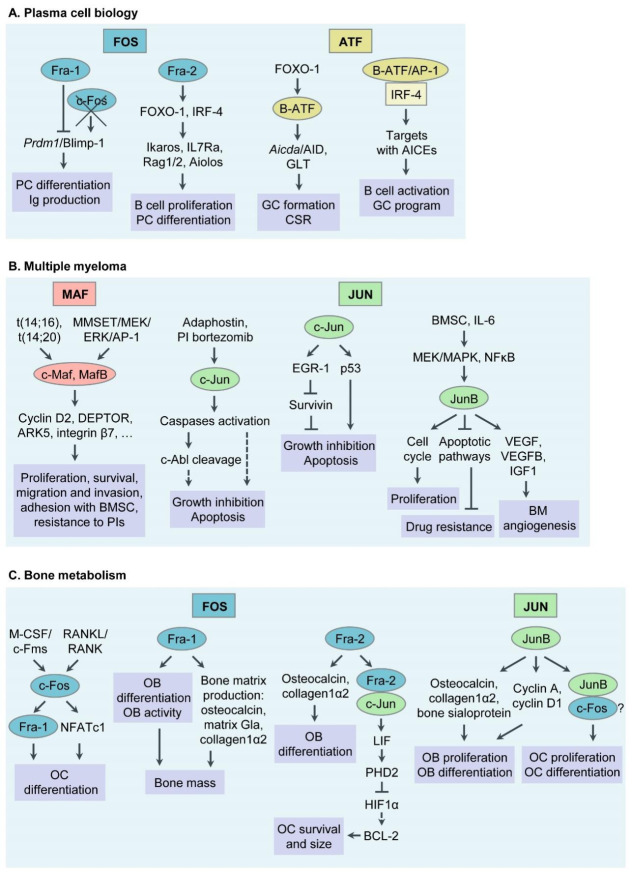
Functions of Activator Protein-1 (AP-1)/JUN, FOS, ATF and MAF transcription factor (TF) subfamily members in plasma cell (PC) biology, multiple myeloma (MM) pathophysiology, bone metabolism and MM associated bone disease. (**A**) Functions of AP-1 TFs in PC biology. (**B**) Functions of AP-1 TFs in MM pathogenesis. (**C**) Functions of AP-1 TFs in bone metabolism and MM associated bone disease. Ig, immunoglobulin; GC, germinal center; CSR, class switch recombination; AID, activation- induced cytidine deaminase; GLT, germline transcript; AICEs, AP-1-IRF composite elements; BM, bone marrow; BMSC, bone marrow stromal cell; PI, proteasome inhibitor; OC, osteoclast; RANKL, receptor activator of NFκB ligand; M-CSF, macrophage colony stimulating factor; NFAT, nuclear factor of activated T cells; LIF, leukaemia inhibitory factor; OB, osteoblast.

AP-1 TFs play a critical role in PC formation and function. When compared to IgM- positive B cells, c-Jun, c-Fos and FosB expression are upregulated in PCs, whereas JunB expression is downregulated and JunD remains unchanged [23]. In B cells, increased expression levels of JunB, JunD, FosB and Fra-1 are detected after stimulation of primary B cells via the B cell receptor (BCR) and/or the CD40 receptor [24]. Moreover, AP-1 TFs c-Jun, JunB, JunD, c-Fos and FosB are induced through activation of Mef-2, a TF associated with B cell development from pre-B cells to immature B cells [25]. Of note, PC differentiation is coupled to division-dependent DNA hypomethylation at CpG loci at enhancer sites; with binding motifs for AP-1 TFs B-ATF, ATF3 and Fra-1 being demethylated first [26]. Specific functions of selected AP-1 TF family members during PC differentiation will be discussed below (Table 1 and Figure 1A).

### 2.1. Fra-1

The Fos-related antigen 1 (Fra-1) reduces proliferation and can induce apoptosis depending on the cellular context. Specifically, Fra-1 suppresses B cell differentiation into PCs. This effect is mediated through inhibition of Blimp-1 expression by direct binding of Fra-1 to the *Prdm1*/Blimp-1 promoter, which thereby prevents binding of c-Fos. Consequently, enhanced PC differentiation and elevated antibody responses occur in mice with B cell-specific deletion of Fra-1. In contrast, Fra-1-overexpressing mice are characterized by impaired PC differentiation and decreased Ig production [27,28,29].

### 2.2. Fra-2

Independent of Fra-1, Fra-2 acts as an enhancer of B cell proliferation and differentiation at multiple stages. Fra-2-deficient mice display decreased B cell numbers in the BM and spleen. Moreover, the in vitro transition of pro-B cell/large pre-B cells from Fra-2-deficient mice to small B cells and immature B cells is impaired. Mechanistically, Fra-2 induces FOXO-1 and IRF-4 gene expression followed by downstream activation of Ikaros, IL7Ra, Rag1/2 and Aiolos [24]. Together with Fra-1, Fra-2 play key regulatory roles in bone metabolism [29] (see below).

### 2.3. B-ATF

B-ATF is a key regulator of GC formation and class switch recombination (CSR). Mechanistically, B-ATF acts as a downstream target of FOXO-1 and regulates CSR by modulating the expression of Activation-induced cytidine deaminase (*Aicda*/AID) and GermLine Transcripts (GLTs) from the Ig locus of B cells in GC. Subsequently, GC B cells are able to differentiate into PCs or memory B cells [30,31,56]. In this context, B-ATF^-/-^ B cells partially phenocopy IRF-4^−/−^ B cells. Of note, due to its low DNA binding affinity IRF-4 forms a heterodimer- complex with B-ATF and binds to the AP-1-IRF composite (AICE) motif (GAAA(NNNN)TGAGTCA), thereby inducing expression of genes involved in B cell activation and the GC response [32,33].

## 3. AP-1 in Multiple Myeloma

Besides acting as critical regulators in PC differentiation, AP-1 TFs are emerging as “master regulators” of aberrant gene expression programs in MM. Below we will discuss functions of AP-1 TFs that have specifically been associated with MM pathogenesis during recent years, c-Maf and MafB, c-Jun, JunB, in particular. Whether Fra-1, Fra-2, B-ATF and other AP-1 family members are deregulated in MM cells is currently unknown and subject of our own and others’ ongoing research efforts (Table 1 and Figure 1B).

### 3.1. c-Maf and MafB

Somatic hypermutation (SHM) and CSR of Igs, two key features of B cell development in GC, are also involved in the ontogeny of MM. Aberrant CSR, V(D)J rearrangement or receptor revision mediated via aberrant expression of AID results in chromosomal translocations, one of the central characteristics of MM. In up to 90% of MM cells, chromosomal translocations affect chromosome 14q32, leading to the placement of various oncogenes under the control of the powerful enhancer of *IGH* genes. Importantly, these oncogenes also include members of the AP-1 TF family, *c-MAF* in t(14;16) (~3–5%) and *MAFB* in t(14;20) (~1.5%), in particular. In addition to t(14;16), c-Maf expression is also triggered by the MMSET/MEK/ERK/AP-1 (c-Fos) signaling pathway (~50%) [34].

By comparing two independent gene-expression profiling studies, 12 deregulated genes have been identified within the molecular “Maf subgroup” (t(14;16)/*c-MAF* or t(14;20)/*MAFB*), including cyclin D2, integrin β7 and ARK5 [57]. c-Maf, in particular, promotes MM cell proliferation via cyclin D2; cell migration and invasion via ARK5; cell survival via DEP domain-containing mTOR-interacting protein (DEPTOR)-dependent activation of the PI3K/AKT pathway; and pathological interactions between BM stroma and MM cells followed by VEGF secretion via integrin β7 [34,35,36,37,57]. These early initiating events define the biological background of MM cells and influence secondary events including copy number changes (chromosome gains/losses), mutations and secondary Ig translocations. Indeed, genetic and microenvironmental alterations impact the likelihood of developing high-risk states of the disease [11,12,15,18,57].

Of note, “poor-prognostic” MM patients with the t(14;16) translocation, unlike other molecular subgroups, are characterized by innate resistance to the proteasome inhibitor (PI) bortezomib. Mechanistically, increased c-Maf protein stability and PI resistance is mediated through the inhibition of Glycogen Synthase Kinase 3 beta (GSK3β) [38]. Similar to c-Maf, PIs bortezomib and carfilzomib also abrogate degradation of MafB protein, which leads to intrinsic resistance to PIs in MM cells with MafB overexpression [39].

Finally, while lytic lesions are pathognomic for MM (occurring in more than 80% of patients), the Maf subtype has a low incidence of bone disease. The lack of bone disease may be, at least in part, explained by c-Maf or MafB-induced osteopontin (OPN) expression by MM cells [58,59] (also see Section 3.4).

### 3.2. c-Jun

Surprisingly, our own and other data have demonstrated that MM patients with low levels of “oncogenic” c-Jun have a shorter overall and event-free survival when compared to patients with normal or high levels of c-Jun. Indeed, drug- induced upregulation of c-Jun inhibits MM cell proliferation and induces apoptosis via caspase-mediated c-Abl cleavage [41] as well as via Early Growth Response protein 1 (EGR-1) [43]. In agreement with these data, logic programming demonstrated a significantly lower c-Jun/Fos activity in MM patients vs. normal controls, regardless of treatment or age [40]. Moreover, PIs bortezomib, carfilzomib and ixazomib induce caspase-dependent cleavage of Myeloid Cell Leukemia-1 (Mcl-1), a pro-survival member of the Bcl-2 family. The resultant Mcl-1^128–350^ fragment translocates into the cell nucleus and triggers MM cell death via induction of c-Jun [42]. Finally, JNK-induced c-Jun binds to the AP-1 binding site of the p53 promoter region and triggers apoptosis by upregulation of p53 [44].

### 3.3. JunB

Recently, we demonstrated for the first time that another AP-1 family member, JunB, plays a pivotal role in MM pathogenesis. Our data showed that a specific and rapid, MEK/MAPK- and NFκB-dependent induction of JunB in MM cells is essential for MM cell proliferation and survival. In addition, JunB protected MM cells against dexamethasone- and PI bortezomib-induced cell death [45]. Our most recent data additionally demonstrated a role for JunB in MM BM angiogenesis. Specifically, we showed that BM-induced expression of JunB was significantly correlated with angiogenic factors VEGF, VEGFB and IGF1 expression and secretion in primary MM cells and cell lines both in vitro and in vivo, and consequently with increased vessel density in patient-derived BM sections [46].

### 3.4. AP-1 in Bone Metabolism and MM Bone Disease

Increased size and number of bone resorbing osteoclasts (OCs) and a decreased activity of osteoblasts (OBs) characterize MM bone disease, a hallmark of MM. Besides c-Maf and MafB, whose expression levels are correlated with a low number of bone lesions in MM, other AP-1 members have been associated with osteolytic bone disease in MM [58,59]. Specifically, c-Fos acts as an essential TF for OC differentiation. The lack of c-Fos results in a block of OC differentiation and in increased numbers of BM macrophages [47,60]. Mechanistically, c-Fos is induced by Macrophage Colony-Stimulating Factor (M-CSF) and Receptor Activator of NFκB Ligand (RANKL) and promotes the expression of Fra-1 and Nuclear Factor of Activated T cells c1 (NFATc1). At the final stage of OC differentiation, NFATc1 cooperates with c-Fos in order to induce OC-specific genes such as TRAP, calcitonin receptor and cathepsin K [48,49,50]. Moreover, the size and survival of OCs is controlled by Fra-2 through Leukaemia Inhibitory Factor (LIF) and hypoxia. Fra-2 transcriptionally induces LIF via Fra-2/c-Jun heterodimers, and modulates LIF/LIF-receptor/PHD2/HIF1α signaling. Fra-2 transgenic mice exhibit osteosclerosis with enhanced bone formation, whereas bones of Fra-2-deficient newborn mice have increased size and numbers of OCs [29,54]. In addition, Fra-2 regulates OB differentiation through transcriptional regulation of osteocalcin and collagen1α2; and Fra-2-overexpressing mice are osteosclerotic [53]. Similarly, another member of Fos proteins, Fra-1, regulates the activity of OBs via the production of bone matrix components, including osteocalcin, collagen1α2 and matrix Gla protein. Mice overexpressing Fra-1 develop osteosclerosis [51,52]. Specifically, both Jun and Fos proteins regulate bone formation and remodeling. JunB is essential for OB proliferation and differentiation. Mice lacking JunB are osteopenic, with reduced cyclin D1 and cyclin A expression, and decreased collagen1α2, osteocalcin and bone sialoprotein production. Moreover, loss of JunB in osteoclast precursors leads to defects in OC proliferation and differentiation, probably through acting as partner of c-Fos [55] (Table 1 and Figure 1C).

MM cells suppress OBs through expression and secretion of the Wnt antagonist sclerostin, an osteocyte-expressed negative regulator of bone formation. Consequently, downregulation of Fra-1, Fra-2 and c-Jun in BM stromal cells cocultured with MM cells in an OB differentiating medium was rescued by an anti-sclerostin monoclonal antibody [61]. Furthermore, bortezomib inhibits OC differentiation and the bone resorption activity of OCs. The mechanisms of inhibition involved in the later phase of differentiation and activation among others occurred through inhibiting AP-1 TFs [62]. These findings indicate that deregulated AP-1 TFs, Fos proteins in particular, play an essential role in the differentiation and activity of OB and OC, and regulate bone metabolism in MM.

## 4. Targeting AP-1 TFs for MM Therapy

Accumulating evidence demonstrates a crucial role of deregulated AP-1 TFs in tumorigenesis in general, and MM in particular. AP-1 TFs therefore represent appealing therapeutic targets. However, TFs have been considered “undruggable” until recently due to their structural disorder (three-dimensional (3D) structure and architecture are very labile and dependent on TF interaction with functional proteins), their lack of tractable active sites (large protein-protein interfaces, lack of deep protein pockets) and their intracellular (often nuclear) localization. Nevertheless, with the progress of our understanding of the biochemical and biological properties of TFs, this paradigm does not hold true any longer. Indeed, members of the AP-1 family have emerged as worldwide actively pursued therapeutic targets, with a potentially high therapeutic index [17,63,64,65]. In MM, our own and other studies suggest therapeutic strategies that inhibit c-Maf or JunB and induce c-Jun activity.

**Figure 2:**
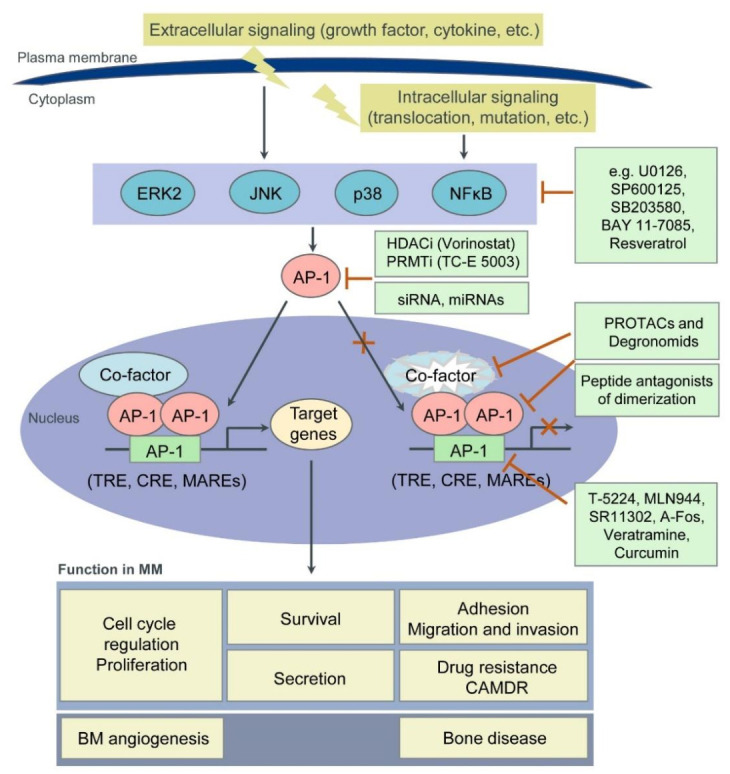
Pathophysiologic functions of AP-1 TFs in MM and derived therapeutic strategies. Intrinsic cellular (translocations, mutations) and intermittent extracellular environmental signals (e.g., growth factors, cytokines) trigger AP-1 TF activity via various signaling pathways including the extracellular-signal regulated kinase (ERK), JUN amino-terminal kinase (JNK), p38 and NFκB. Target genes of AP-1 TFs play a pivotal role in MM pathogenesis mediating tumor cell proliferation, adhesion, migration and invasion, apoptosis, survival and drug resistance as well as BM angiogenesis. Besides inhibiting TF expression by siRNAs or miRNAs, novel approaches to target TFs in general, and AP-1 TFs in particular, include: disrupting the interaction of TFs with either functionally critical protein binding partners (e.g., by peptide antagonists of dimerization) or the DNA (by oligodeoxynucleotide decoys, pyrrole-imidazole polyamides or small molecules); modulating the epigenetic events through DNA methylation, histone methylation or modification (e.g., by histone deacetyltransferases inhibitors (HDACi) or protein arginine methyltransferases inhibitors (PRMTi)); inducing proteasomal degradation of TFs by altering their ubiquitylation, as well as by utilizing PROteolysis-TArgeting Chimaeras (PROTACs) or Degronomids; and inhibiting TF expression by modulating their regulators (i.e., MAPK- or NFκB- signaling molecules).

Besides inhibiting their expression (i.e., by siRNAs, miRNAs), novel approaches to target TFs in general, and AP-1 TFs in particular, include: (1) the disruption of either their interaction with functionally critical protein binding partners or; (2) their binding to the DNA (oligodeoxynucleotide decoys, pyrrole-imidazole polyamides or small molecules); (3) the modulation of their epigenetic binding through DNA methylation, histone methylation or modification; (4) the induction of proteasomal degradation of TFs by altering their ubiquitylation; as well as by utilizing PROteolysis-TArgeting Chimaeras (PROTACs) or Degronomids; (5) the inhibition of TF expression by modulating their regulators (i.e., MAPK- or NFκB-signaling molecules); (6) the use of reversible covalent drugs directed against non-conserved cysteines; and (7) the modulation of TF auto-inhibition. Moreover, disordered regions within TFs, which become structured upon interaction with binding partners (“coupled folding and binding”) may also represent attractive therapeutic targets. Indeed, these regions have a higher proportion of potential cavities and can more easily adjust to small molecules [65,66,67,68,69]. Below we discuss some potential approaches to target AP-1 TFs in MM (Figure 2 and Table 2).

### 4.1. Targeting Protein-Protein Interaction

Based on the secondary structure and dimerization properties of the leucine zipper domain of c-Maf, potential peptidic c-Maf dimerization inhibitors were computationally designed. These peptide inhibitors are able to interact selectively with the c-Maf leucine zipper, thereby affecting the degree of their structural organization and destabilizing homodimers [66,70].

In addition, many efforts have been made to search for peptides which exhibit high affinity for the leucine zipper dimerization domains of c-Jun or c-Fos and to inhibit these bZIP proteins by preventing the formation of functional c-Jun homodimers and c-Jun: c-Fos heterodimers [71,72,73,74,75,76,89]. Excitingly, an anti-Jun and anti-Fos superzipper has been demonstrated to bind to both the c-Jun and c-Fos leucine zipper peptides [77]. Finally, pharmacological inhibition of transcriptional co-factors of AP-1 TFs, such as CBP/p300, may represent yet another strategy to attenuate AP-1 activity [17].

### 4.2. Targeting Protein-DNA Interaction

Informed by the x-ray crystal structure of the bZIP domain of the AP-1-DNA complex, 3D pharmacophore modeling led to the design and synthesis of T-5224 and its analogues. Specifically, T-5224 inhibits the DNA binding activity of c-Fos: c-Jun without affecting DNA-binding of other TFs or the expression levels of Fos members. Excitingly, T-5224 has been investigated in phase II clinical trials [63,78,79].

Similarly, MLN944 (XR5944) is a DNA binding compound that interacts with the 5′-ATGCAT-3′ palindromic sequence through its two phenazine rings to induce a right-handed twist of the DNA helix. It thereby inhibits c-Jun-DNA binding to the AP-1 TRE site. However, MLN944 also modulates the estrogen receptor alpha-DNA binding on the estrogen response element [80,90]. A new class of conformationally restricted synthetic retinoids has been found to selectively inhibit AP-1 TRE activity without activating the retinoic acid response element [81]. SR11302, an AP-1 inhibition-specific retinoid, displayed antitumor effects in vivo, and represents an important research tool compound [82].

Besides small molecule inhibitors, the peptidic inhibitor A-Fos forms heterodimers with the whole bZIP domain of c-Jun through leucine zipper and an acidic extension, and thereby obstructs binding of c-Jun: A-Fos to the DNA [83].

### 4.3. Epigenetic Inhibitors

Epigenetic alterations activate or suppress AP-1 activities and offer the opportunity to selectively target AP-1 transcriptomes [68].

The acetylation state of lysine residues in histones is determined by the balance between histone deacetyltransferases (HDACs) that remove acetyl groups and histone acetyltransferases (HAT) that transfer acetyl groups, which is critical for regulating gene transcription. HDAC inhibitors (HDACis) including valproic acid (VPA), vorinostat (SAHA), trichostatin A (TSA) and LBH589 suppress the transcription of both c-Jun and Fra-1 and thereby reduce c-Jun: Fra-1 heterodimer formation and activity [84], which may, at least in part, explain their anti-MM activity. Panobinostat, an oral HDACi, has been approved by US FDA for the treatment of MM in patients who have received at least two prior standard therapies [91].

In addition, protein arginine methyltransferases (PRMTs) are ‘writers’ of arginine methylation in histone and non-histone proteins and are involved in aberrant epigenetic networks in cancers. The selective PRMT1 inhibitor TC-E 5003 (TC-E) downregulates the nuclear translocation of c-Jun as well as of NFκB subunits p65 and p50, and directly regulates c-Jun gene expression following lipopolysaccharides (LPS) treatment [68,85].

Of note, novel technologies enhance our understanding of the epigenetic impact on TF binding to the DNA. For example, utilizing Selective Microfluidics-based Ligand Enrichment followed by sequencing (SMiLE-seq), a novel semi-automated protein-DNA interaction characterization technology, recently resulted in a de novo motif discovery on all Jun: Fos heterodimers. It thereby provided novel insights into partner- specific heterodimer DNA-binding preferences [92]. Moreover, c-Jun: c-Fos selectively binds to DNA sequence motifs with methylated CpG residues (meAP-1), thereby reversing epigenetic silencing [92,93]. Conversely, the anti-MM activity of small molecule inhibitors of DNA methyltransferase (DNMT), such as 5-azacytidine, may be, at least in part, explained by inhibition of c-Jun: c-Fos binding [94].

### 4.4. TF Degradation

By demonstrating that glucocorticoids increase ubiquitination-dependent degradation of c-Maf [95], early studies already indicated the therapeutic potential of TF degradation. Moreover, c-Maf and MafB phosphorylation followed by destabilization and degradation is mediated by the Ser/Thr kinase Glycogen Synthase Kinase 3 (GSK3) [96].

Representing a new treatment technology, PROteolysis Targeting Chimeras (PROTACs) and Degronomids hijack E3 ubiquitin ligases (e.g., Von Hippel-Lindau (VHL), cereblon (CRBN), Inhibitor of Apoptosis Proteins (IAPs), and Murine Double Minute 2 (MDM2)) for selective protein ubiquitination and subsequent degradation by the proteasome [97,98]. Specifically, PROTACs and Degronomids contain a small molecule or peptide as a ligand to recruit the E3 ubiquitin ligase, a ligand to bind the protein of interest (POI), and a linker connecting the two ligands to ensure optimal interaction of the E3 ligase and the POI [97]. To date over forty target proteins have been specifically degraded by PROTACs. Recent data have demonstrated great potential of PROTACs to target “difficult-to-target” proteins, including TFs and transcription regulating proteins, such as nuclear receptors and Bromodomain and ExtraTerminal (BET) proteins [98,99].

In MM, IMiD-based CRBN-targeted and VHL-targeted PROTACs (i.e., ARV-825 and ARV-763, respectively), which degrade the BET-domain-containing protein BRD-4 induced cell cycle arrest and apoptosis and overcame drug resistance in pre-clinical models of MM [100,101,102,103,104].

Additional PROTACs that are directed against a multitude of other “difficult-to-target” proteins including AP-1 TFs are under development. Ongoing efforts aim to improve the pharmacokinetics, bioavailability and tissue distribution of PROTACs; as well as to identify suitable E3 ubiquitin ligases for these molecules to target specific proteins [105].

Of note, both the E3 ubiquitin ligase HERC4 as well as TMEPAI mediate c-Maf ubiquitination and proteasomal degradation, thereby inhibiting MM growth. Further supporting a key role for c-Maf in MM progression, HERC4 as well as TMEPAI expression levels steadily decrease during disease progression [106,107]. Therefore, therapeutic strategies that restore functional HERC4 and TMEPAI expression may represent promising therapeutic strategies for MM therapy. In contrast, the deubiquitinase USP7 stabilizes Maf proteins and promotes MM cell survival. Therefore, targeting the USP7/Maf axis may represent another potential strategy for MM therapy [108].

### 4.5. Natural Products

Although underlying mechanisms are not fully understood, several natural products modulate AP-1 TF activity and exhibit anti-tumor effects (reviewed in [109]). For example, the anti-MM activity of curcumin but also resveratrol (trans-3,4′,5-trihydroxystilbene) may be, at least in part, explained by their ability to inhibit c-Fos and c-Jun expression, heterodimer formation and DNA binding [86,87]. Moreover, veratramine, an alkaloid derived from Veratrum plants, has been identified as a potent natural modulator of AP-1, which selectively binds to TRE and regulates AP-1-dependent gene transcription [88].

## 5. Conclusions

AP-1 TFs play essential roles in the transcriptional control of GC B cell development and PC differentiation. Dysregulation of AP-1 is an important mechanism in the oncogenic transformation and drug resistance of MM. Although recent discoveries are exciting, the therapeutic exploration of AP-1 TFs has just begun. Continuing basic and translational research on AP-1 TFs utilizing new technologies such as NMR-based screens, differential scanning fluorimetry (DSF), in silico 3D modelling, as well as Slim-seq will be fundamental to further advance our insights on the complex function of this TF family, facilitating the identification of valuable targets and the development of derived innovative therapies for MM to once more improve patient outcome.

## Figures and Tables

**Table 1:** Function of AP-1 in plasma cell biology and multiple myeloma pathophysiology.

AP-1 Member	Activity	Mechanism	References
**Plasma cell biology**			
Fra-1	Suppresses B cell differentiation into PCs and decreases Ig production	Inhibition of *Prdm1*/Blimp-1 expression by preventing binding of c-Fos to the promoter	[27,28,29]
Fra-2	Enhances B cell proliferation and differentiation at multiple stages	Transcriptional induction of FOXO-1 and IRF-4 expression, and their downstream targets Ikaros, IL7Ra, Rag1/2 and Aiolos	[24]
B-ATF	Essential for GC formation and effective CSR	Downstream of FOXO-1, modulating the expression of *Aicda*/AID and GLTs from the Ig locus of B cells in the GC	[30,31]
	Regulates B cell activation and GC response	Binding of B-ATF containing AP-1 complexes and IRF-4 to the AICE motif of target genes	[32,33]
**Multiple myeloma**			
c-Maf MafB	Overexpressed in MM	Chromosomal translocation t(14;16), t(14;20) MMSET/MEK/ERK/AP-1 signaling sequelae	[11,18,34]
	Promote MM cell proliferation, migration and invasion, survival, adhesion and pathological interactions with BMSC	Regulation of cyclin D2, ARK5, DEPTOR, and integrin β7 expression	[35,36,37]
	Confer resistance to PIs bortezomib and carfilzomib	Abrogation of GSK3β-mediated proteasomal degradation of c-Maf and MafB	[38,39]
c-Jun	Lower expression in primary MM cells compared to normal PCs	Unknown	[40]
	Upregulated in MM cells by adaphostin or bortezomib Inhibits proliferation and induces apoptosis	Caspase-mediated c-Abl cleavage Upregulation of EGR-1 Upregulation of p53	[41,42,43,44]
JunB	BMSC- and IL-6- triggered upregulation in MM cells	MEK/MAPK- and NFκB- dependent	[45]
	Promotes MM cell proliferation	Cell cycle regulation	
	Protects MM cells against dexamethasone- and bortezomib- induced cell death	Inhibition of apoptotic pathways	
	Promotes MM BM angiogenesis	Transcriptional regulation of angiogenic factors VEGF, VEGFB and IGF1	[46]
**Bone metabolism**			
c-Fos	Regulates OC differentiation (Block in OC differentiation in mice lacking c-Fos)	Induced by RANKL and M-CSF Transcriptional regulation of Fra-1 and NFATc1	[47,48,49,50]
Fra-1	Regulates OB activity and bone matrix formation (Mice overexpressing Fra-1 develop osteosclerosis)	Regulation of bone matrix component production by OBs (osteocalcin, collagen1α2, and matrix Gla protein)	[51,52]
Fra-2	Regulates OB differentiation (Fra-2-overexpressing mice are osteosclerotic)	Transcriptional regulation of osteocalcin and collagen1α2	[53]
	Controls OC survival and size (Increased size and numbers of OCs in Fra-2-deficient mice)	Transcriptional induction of LIF via Fra-2: c-Jun heterodimers Modulation of LIF/LIF-receptor/PHD2/HIF1α signaling sequelae	[54]
JunB	Regulates OB proliferation and differentiation (Mice lacking JunB are osteopenic)	Cyclin D1 and cyclin A expression, and collagen1α2, osteocalcin and bone sialoprotein production	[55]
	Regulates OC proliferation and differentiation	Dimerization partner of c-Fos (?)	

Abbreviations: PCs, plasma cells; Ig, immunoglobulin; GC, germinal center; CSR, class switch recombination; AID, activation- induced cytidine deaminase; GLTs, germline transcripts; AICE, AP-1-IRF composite element; MM, multiple myeloma; BM, bone marrow; BMSC, bone marrow stromal cells; PIs, proteasome inhibitors; OC, osteoclast; RANKL, receptor activator of NFκB ligand; M-CSF, macrophage colony stimulating factor; NFAT, nuclear factor of activated T cells; LIF: leukaemia inhibitory factor; OB, osteoblast.

**Table 2:** Potential strategies to target AP-1 and candidate inhibitors.

Strategies	Inhibitors	Targets	References
Inhibition of protein-protein interactions	Peptidic inhibitors of c-Maf dimerization	Leucine zipper motif of c-Maf	[70]
	Peptide antagonists of c-Jun dimerization	Leucine zipper motif of c-Jun	[71,72,73,74]
	Peptide antagonists of c-Jun: c-Fos dimerization	Leucine zipper motif of c-Jun or c-Fos	[75,76]
	Leucine zipper peptide (Superzipper)	Leucine zipper dimerization domains of both c-Jun and c-Fos	[77]
Inhibition of protein- DNA binding	T-5224	bZIP domain of c-Fos/AP-1 -DNA complex	[78,79]
	MLN944 (XR5944)	TRE	[80]
	SR11302	TRE	[81,82]
	Dominant negative peptide A-Fos	bZIP domain of c-Jun	[83]
Regulation of epigenetic events	Valproic acid (VPA) Vorinostat (SAHA) Trichostatin A (TSA) LBH589	HDAC (Transcriptional suppression of c-Jun and Fra-1 expression)	[84]
	TC-E 5003 (TC-E)	PRMT (Suppression of c-Jun expression and nuclear translocation)	[85]
Natural products	Curcumin	Suppression of c-Fos and c-Jun expression and their binding to DNA	[86]
	Resveratrol	Suppression of c-Fos and c-Jun expression and AP-1 activity	[87]
	Veratramine	TRE	[88]

Abbreviations: bZIP, basic leucine zipper; TRE, TPA-response element; HDAC, histone deacetyltransferases; PRMT, protein arginine methyltransferases.
